# Enhanced vehicle routing for medical waste management via hybrid deep reinforcement learning and optimization algorithms

**DOI:** 10.3389/frai.2025.1496653

**Published:** 2025-02-12

**Authors:** Norhan Khallaf, Osama Abd-El Rouf, Abeer D. Algarni, Mohy Hadhoud, Ahmed Kafafy

**Affiliations:** ^1^Machine Learning Department, Faculty of Artificial Intelligence, Menoufia University, Menoufia, Egypt; ^2^College of Computer and Information Sciences, Princess Nourah Bint Abdulrahman University, Riyadh, Saudi Arabia; ^3^Faculty of Computer and Information, Menoufia University, Menoufia, Egypt; ^4^Data Science Department, Faculty of Artificial Intelligence, Menoufia University, Menoufia, Egypt

**Keywords:** closed capacity vehicle routing problem, Q learning, DQN, medical waste routing optimization, unsupervised learning algorithm, hybrid optimization algorithms, multi-homogeneous agent systems

## Abstract

Modern technologies, particularly artificial intelligence, play a crucial role in improving medical waste management by developing intelligent systems that optimize the shortest routes for waste transport, from its generation to final disposal. Algorithms such as Q-learning and Deep Q Network enhance the efficiency of transport and disposal while reducing environmental pollution risks. In this study, artificial intelligence algorithms were trained using Homogeneous agent systems with a capacity of 3 tons to optimize routes between hospitals within the Closed Capacitated Vehicle Routing Problem framework. Integrating AI with pathfinding techniques, especially the hybrid A*-Deep Q Network approach, led to advanced results despite initial challenges. K-means clustering was used to divide hospitals into zones, allowing agents to navigate the shortest paths using the Deep Q Network. Analysis revealed that the agents’ capacity was not fully utilized. This led to the application of Fractional Knapsack dynamic programming with Deep Q Network to maximize capacity utilization while achieving optimal routes. Since the criteria used to compare the algorithms’ effectiveness are the number of vehicles and the utilization of the total vehicle capacity, it was found that the Fractional Knapsack with DQN stands out by requiring the fewest number of vehicles (4), achieving 0% loss in this metric as it matches the optimal value. Compared to other algorithms that require 5 or 7 vehicles, it reduces the fleet size by 20 and 42.86%, respectively. Additionally, it maximizes vehicle capacity utilization at 100%, unlike other methods, which utilize only 33 to 66% of vehicle capacity. However, this improvement comes at the cost of a 9% increase in distance, reflecting the longer routes needed to serve more hospitals per trip. Despite this trade-off, the algorithm’s ability to minimize fleet size while fully utilizing vehicle capacity makes it the optimal choice in scenarios where these factors are critical. This approach not only improved performance but also enhanced environmental sustainability, making it the most effective and challenging solution among all the algorithms used in the study.

## 1 Introduction

Medical waste management is a crucial concern for hospitals and healthcare facilities, as it involves handling a wide range of materials that can harm human health and the environment if not properly managed (Fawkia et al., 2019). Medical waste is classified into two types: hazardous and non-hazardous. Effective management requires stringent practices and protocols to prevent the spread of infections, environmental contamination, and harm to healthcare workers (Attrah et al., 2022). These practices include sorting waste into various types of bags, utilizing specialized vehicles to transport it to disposal sites without leaking harmful substances, securely storing it temporarily before final disposal, and employing methods such as incineration in specialized facilities or waste burial (Zhao et al., 2021). Artificial intelligence aims to develop systems capable of performing tasks that traditionally require human intelligence (Stahl, 2021). It significantly contributes to healthcare by devising methods to train agents (vehicles) and create strategic plans for the safe transportation of medical waste from hospitals to disposal sites via the shortest routes possible, while also considering the available capacity of the agents. This challenge is known as the Closed Capacity Vehicles Routing Problem (CCVRP), which falls under the category of NP-hard problems, as agents are required to return to the depot (collection center) (Sluijk et al., 2023). There are two main types of Capacity Vehicle Routing Problems: closed and open. In the closed CVRP, each route originates and terminates at the same depot, necessitating the return of vehicles to the depot after serving all hospitals (Borcinova, 2017).

In contrast, the open CVRP allows routes to terminate at any hospital location without the need to return to a central depot. Machine Learning, a branch of Artificial Intelligence, focuses on developing models that enable systems to learn from data and improve performance over time. It encompasses various learning paradigms, including Supervised, Unsupervised, Semi-supervised, and Reinforcement Learning (RL).

RL is a branch of machine learning where an agent learns to make optimal decisions by interacting with an environment and receiving feedback in the form of rewards. Unlike supervised learning, which trains models on fixed datasets with labeled examples, RL focuses on learning from the outcomes of actions through a trial-and-error process. This approach is particularly effective for complex decision-making tasks where the optimal strategy is not immediately apparent (Farhaoui, 2024).

In the context of the CVRP, RL is highly advantageous as it optimizes routes and resource allocation in dynamic, large-scale scenarios. By training agents to explore various routes and adapt to changing conditions, RL can significantly reduce operational costs, enhance efficiency, and respond to real-time changes. RL methods include Model-Free RL, where the agent learns solely through trial and error without relying on an explicit model of the environment, and Model-Based RL, where the agent constructs a model for simulation and planning to inform its decisions (Mazyavkina et al., 2021). Deep Reinforcement Learning (DRL) extends traditional RL by incorporating deep learning techniques, such as Deep Q-networks (DQN), to address more complex tasks effectively. DRL offers a robust framework for tackling challenging problems and achieving superior performance in dynamic environments.

A fundamental concept in RL is the policy, which governs the agent’s actions in any given state. The policy essentially maps states or observations of the environment to the actions the agent should take to maximize cumulative rewards. Policies can be categorized into two main types: on-policy and off-policy. In on-policy learning, the agent learns and improves the policy it is currently using, with examples including SARSA and policy gradient algorithms. In off-policy learning, the agent learns the value of a different policy, allowing it to leverage historical data or experiences, which can be more efficient and flexible, especially in scenarios where exploring a new policy might be risky or costly. Examples of off-policy algorithms include Q-learning (QL) and DQN (Dong et al., 2020).

The proposed solution in our paper involves the use of Q-learning, DQN, and a hybrid approach that integrates A* with DQN. Additionally, a strategy is introduced where hospitals in the case study are divided into different zones, with DQN applied within these zones to determine the shortest route using a cluster DQN approach. To further optimize the utilization of each agent’s total capacity, fractional knapsack dynamic programming combined with DQN will be employed. This approach provides an optimal solution to this NP-hard problem.

Q-learning is a machine learning algorithm used in reinforcement learning (RL). In Q-learning, the agent learns a policy to take actions in the environment that maximize cumulative rewards over time. Here’s a basic explanation of how Q-learning works: it involves states (S), actions (A), rewards (R), a Q-table, and the balance between exploration and exploitation. Over time, as the agent interacts with the environment and updates its Q-table, it learns the best actions to take in each state to maximize the expected cumulative reward. Q-learning is widely used in applications like robotics, game-playing, and autonomous systems, where agents learn optimal decision-making policies through trial and error (Clifton and Laber, 2020).

To enhance results, multi-agent training and testing will be employed using neural networks to select the shortest route through the DQN approach. DQN utilizes deep neural networks to approximate the Q-value function (Farhaoui, 2024). This function estimates the expected cumulative rewards for taking specific actions in a given state. By iteratively updating its Q-values based on experiences from interacting with the environment, DQN learns to optimize routing decisions for vehicles. Leveraging deep learning techniques, DQN effectively handles the complex decision-making process involved in solving CVRP (Hou et al., 2023).

To further refine the results, the hybrid A* approach is integrated with DQN. A* (pronounced “A-star”) is a widely used pathfinding algorithm designed to determine the shortest path between nodes in a graph. It blends uniform cost search and greedy best-first search (Laparra, 2019). A* assesses nodes by weighing the cost to reach the node from the start and an estimation of the cost to reach the goal from the node, typically using a heuristic function. In the context of CVRP, A* efficiently explores potential routes for vehicles, considering variables such as distance, capacity constraints, and time (Birkenes, 2023).

Additionally, alternative methods are explored to achieve optimal solutions for CVRP. One approach hybridizes K-means clustering with DQN, while another incorporates fractional knapsack dynamic programming with DQN (Zhu, 2022). The K-means clustering technique partitions a dataset into “k” distinct, non-overlapping clusters based on data point similarities (Rahul et al., 2023). Within each cluster, the shortest route between nodes is selected using the DQN approach.

These algorithms significantly enhance medical waste management in large-scale hospitals, ensuring safe transport to waste disposal centers. However, conventional algorithms either collect all waste or none, based on agent capacity. This limitation often results in underutilized vehicle capacity. In contrast, the Fractional Knapsack Dynamic Programming approach allows agents to collect partial or full waste amounts based on available capacity. This ensures maximum utilization of each agent’s capacity with no wasted space in the vehicles (Piedra de la Cuadra, 2023).

Fractional Knapsack Dynamic Programming addresses the problem of maximizing the total value of items in a knapsack, allowing fractional inclusion of items. Unlike the 0/1 knapsack problem, which requires full inclusion or exclusion of items, the fractional version enables partial inclusion. The solution involves sorting items by their value-to-weight ratio and selecting the most valuable items or fractions until the knapsack’s capacity is reached (Frank et al., 2024).

This approach is typically solved using a greedy algorithm, which efficiently ensures the optimal distribution of value given the weight constraints. The Main challenge in this research was training and testing agents using DQN and then integrating them with other algorithms for managing medical waste across a set of hospitals in the Menoufia Governorate. The results revealed differences among various combinations, with each algorithm offering specific advantages based on criteria such as finding the shortest path between hospitals and reducing time, distance, cost, and the number of agents needed. The research did not only focus on minimizing costs but also included a comparison between fuel-powered agents and battery-operated agents to determine the most cost-effective and environmentally friendly option. The main contribution of this research lies in:

1. Novel Application of Algorithms: The research applies algorithms to train vehicles for selecting the shortest routes between government hospitals in Menoufia Governorate, Egypt, to collect and dispose of medical waste. The system enhances agent decision-making through reinforcement learning algorithms.
2. Integration of Deep Reinforcement Learning and Optimization: By combining deep reinforcement learning with optimization algorithms, the study seeks to improve solution quality and achieve optimal results. Additionally, integrating the fractional knapsack problem ensures full vehicle capacity utilization, leading to increased economic efficiency and reduced environmental impact by decreasing the number of vehicles required.
3. Reduction in Labor Costs: The reduction in the number of vehicles directly lowers the need for workers, thus reducing wage expenses.
4. Electric Vehicle Simulation: The research includes a simulation of converting vehicles to electric models to evaluate the potential economic impact of transitioning to electric-powered vehicles.

Despite significant advancements in vehicle routing algorithms and medical waste management strategies, current studies often fail to integrate reinforcement learning techniques and optimization methods that fully address the challenges of the Capacitated Vehicle Routing Problem (CCVRP), especially in large-scale scenarios with strict capacity and environmental constraints. This research aims to bridge this gap by proposing a novel hybrid approach to optimize routes, reduce operational costs, and enhance environmental sustainability. The optimization process involves initially using exact or greedy algorithms (non-learning algorithms), followed by improving the output (such as the shortest route) using reinforcement learning algorithms such as Q-learning, DQN, or SARSA. This integration of traditional methods with reinforcement learning techniques helps develop effective applications for managing and solving environmental and economic crises in fields such as healthcare, agriculture, industry, and others. This integrated solution is validated within the case study of the Menoufia Governorate, achieving superior performance metrics, including reduced agent deployment and optimized fuel consumption. By addressing these limitations, the research bridges the identified gaps and establishes a scalable, efficient, and environmentally friendly framework for medical waste management. The research paper presents a comprehensive analysis of these aspects, with Section 2 defining the problem, Section 3 explaining the set of algorithms used, Section 4 detailing the results, Section 5 discussing the algorithms, and Section 6 presenting the conclusions.

## 2 Related work

Most of the previous research that relies on AI and agent training using the reward and punishment approach, or the integration of AI algorithms with other algorithms such as heuristic search algorithms or greedy algorithms in medical waste management, has not been sufficiently available in the published literature. This problem requires the agent to manage the process of collecting waste from hospitals according to its capacity and disposing of it in waste centers while achieving the shortest possible route. This problem is known as CVRP. Below are some studies that have contributed to addressing this issue.

In this study (Bozanta et al., 2022), the problem involves optimizing revenue in a food delivery service with a limited number of couriers using a Markov decision process model. Three approaches were tested: simplifying to a single courier with Q-Learning, using Double Deep Q-Networks (DDQN) for a single courier, and applying DDQN to an extensive model with multiple couriers and orders. The results indicated that Q-Learning for a single courier achieved higher rewards than a rule-based policy. DDQN outperformed both Q-Learning and the rule-based approach, though its effectiveness was dependent on hyper-parameters. The study has several limitations. The Q-learning algorithm struggled with large state spaces, necessitating approximation methods like single-courier models and DDQN. DDQN’s performance was highly sensitive to hyper-parameters. The model only included courier and order locations, missing factors like working time and order preparation time. Additionally, the number of couriers was limited to control the state space size.

Reference study (Yue et al., 2024), the focus is on the time-dependent green vehicle routing problem with time windows, which is more complex than traditional vehicle routing problems due to its simultaneous consideration of transportation time, carbon emissions, and customer satisfaction under time-dependent conditions. The proposed solution involves a multi-objective optimization algorithm that integrates a learnable crossover strategy and an adaptive search strategy based on reinforcement learning. This approach is implemented in two stages: the first stage uses a hybrid initialization strategy to generate high-quality initial solutions and employs crossover strategies to enhance convergence and explore the solution space. The second stage involves the adaptive search for further learning and refinement. Experimental results indicate that this approach achieves better solution quality compared to existing methods, demonstrating superior convergence and diversity. The research has several limitations. The model focuses on a multi-objective optimization problem but does not fully address production scheduling alongside vehicle scheduling. It also lacks consideration of more realistic constraints, such as combined pick-up and delivery needs of customers. Additionally, the model does not account for dynamic changes in the problem, which could be crucial for real-world applications. Future improvements are needed to address these aspects for a more comprehensive solution. The study (Boudanga and Medromi, 2023) addressed in this study is the complex task of managing medical waste, which requires effective strategies to reduce health risks, comply with regulations, and minimize environmental impact. The study proposes a novel approach that utilizes advanced technologies and collaboration to enhance waste management. This approach includes the use of colored bags with identification tags, smart containers with sensors, GPS-equipped vehicles, and outsourced waste treatment. Additionally, it incorporates explainable artificial intelligence (XAI) technologies and genetic algorithms to optimize waste sorting, storage, treatment, and vehicle routing. The results show that integrating these technologies leads to an efficient and intelligent medical waste management system, improving public health and environmental outcomes. The article’s main limitations include a focus primarily on vehicle routing models and cross-docking techniques, potentially overlooking other crucial aspects of medical waste management. While it highlights the use of smart cross-docking centers and specialized treatment centers, it may not fully address the practical challenges of implementing these technologies at scale. The reliance on statistical tests like ANOVA and Tukey may not capture all nuances of system performance. Furthermore, although IoT and XAI technologies are used, their real-world effectiveness and scalability remain uncertain. Future research could benefit from exploring additional optimization strategies and advanced AI techniques to address these gaps.

The study (Khallaf et al., 2024) addressed in the study is the complex issue of routing vehicles with limited capacity for collecting and transporting hazardous medical waste from multiple hospitals to a disposal site. To solve this, various techniques were employed, including reinforcement learning with the SARSA algorithm, Dijkstra’s algorithm, knapsack dynamic programming, and hybrid approaches combining these methods. The results indicate that the hybrid approach of SARSA and knapsack dynamic programming was the most effective. It reduced the number of vehicles required, maximized vehicle capacity, and identified the shortest routes between hospitals, ultimately lowering transportation costs and improving the efficiency of medical waste management. This study faces several limitations. First, the SARSA algorithm, while useful, may not always provide accurate results, particularly for large-scale problems or when dealing with a higher number of hospitals. Additionally, the vehicle routing problem with fixed capacities and specific routes may not be fully addressed by SARSA alone. The study suggests exploring alternative AI methods, such as deep neural networks and advanced reinforcement learning techniques like Double DQN and deep learning approaches. Furthermore, incorporating time windows for hospital visits and developing models with varying vehicle capacities and clustered zones could enhance the system’s effectiveness and flexibility.

The study (Kapadia and Mehta, 2023) addresses the challenges of efficient waste management in urban areas, including increasing waste generation, high transportation costs, and environmental impact due to inefficient collection systems. The proposed solution utilizes IoT-enabled smart bins and advanced algorithms, such as SC-KNN clustering for grouping bins and the Op-A* heuristic for dynamic route optimization. By leveraging real-time data from smart bins, the system minimizes unnecessary trips, reduces fuel consumption, and lowers transportation costs, contributing to environmental sustainability. However, the study highlights limitations, including scalability challenges with larger datasets, integration issues with real-world variables like traffic, and potential inefficiencies in clustering methods. Future work focuses on enhancing the system through predictive models, improved heuristics, and broader validation across diverse geographic contexts. This research demonstrates the potential of IoT and intelligent algorithms in creating sustainable and cost-effective waste management systems.

This study (Karimi et al., 2024) addresses the challenges of managing hazardous medical waste (HMW) during the COVID-19 pandemic, which led to a significant increase in infectious waste volumes, posing risks to public health and the environment. To tackle these issues, a reverse logistics supply chain network is proposed, incorporating temporary treatment centers (TTCs), robust possibilistic programming, and multi-objective optimization techniques such as goal attainment and Lp-metrics. The model optimizes waste allocation, reduces exposure risks, and improves treatment efficiency, validated through a real-case application in Tonekabon City, Iran. Despite its effectiveness, the study faces limitations related to data uncertainty, cost constraints, and environmental trade-offs from certain treatment technologies. Future work suggests integrating advanced waste treatment methods, developing dynamic models with real-time data, validating the approach in diverse regions, and refining sustainability metrics. This research provides a comprehensive framework for addressing medical waste management challenges during pandemics with a focus on sustainability and risk mitigation.

## 3 Problem definition

In this section, the specific issue is defined, focusing on evaluating the most efficient and shortest route for the disposal of hospital waste in Menoufia Governorate. Hospital waste is collected and disposed of two to three times a week, depending on the volume of waste, services provided, and the area of the hospitals. A sample of extracted waste from the first week of January 2024 is used to train our mathematical model. This model aims to find the optimal solution for planning the transportation of waste to disposal sites via the shortest path. The goal is to develop a model that can be used internationally for any waste management CVRP. The Ministry of Health enters into agreements to hire several agents from transportation companies to transport waste from hospitals two or three times a week each month. It should be noted that these agents have their work during the rest of the week not covered by the contract. The cost is calculated based on the rental period specified in the contract. These wastes are considered hazardous, carrying pollutants and diseases. Therefore, they must be disposed of safely, away from residential areas. The waste disposal site is located in Kafr Dawood (node D) in the city of Sadat. The waste transport vehicles depart from the central collection center known as the Health Affairs Center (node C) in Menoufia Governorate. The path of any agent starts from the collection center, then selects the shortest route between hospitals, proceeds to the disposal site, and finally returns to the starting point. This is classified as a Closed CVRP. The hospitals are situated at different indices and locations on Google Maps, as discussed in reference (Khallaf et al., 2024). The geographical positions of each hospital on the map. For example, Node 1, corresponding to Quwisna General Hospital, is located at coordinates (30.55 and 31.13) on the x and y axes, respectively. This study focuses on a sample comprising 15 government and fever hospitals situated in the Menoufia Governorate. Defining the objective of calculating the shortest route connecting a group of hospitals, considering waste disposal capacities, the shortest distance, and time, allows us to classify the problem as a CVRP. CVRP is a logistical challenge that involves optimizing the transportation of wastes using a fleet of vehicles, each with a limited capacity of 3 tons. It is characterized as a directed graph $G=\left(N,A\right)$ , where $N=\left\{0,1,\;\ldots ,n\right\}$ constitutes a set of nodes, and $A=\left\{\left(i,j\right)\in N\times N,i\neq j\right\}$ forms a set of arcs. Node 0 (Node C) denotes the collection center (depot) for a fleet of V agents (vehicles) with identical capacity Q. Node 16 (Node D) signifies the disposal site where waste is managed, while the remaining N nodes represent hospitals with the demand $d_{i}$ , $i\in N-\left\{0,16\right\}$ . Every arc $\left(i,j\right)\in A$ is associated with a positive travel cost, which is represented by the distance between hospitals and denoted as $C_{ij}$ $\mathrm{over}\;\mathrm{arc}\;\left(i,j\right)\in A$ . The following is a mathematical formulation for CVRP (Equations 1–7) (Tirkolaee et al., 2021):


(1) $$\min\;\underset{i,j\in A}{\sum }c_{ij}\;X_{ij}+dis_{ij}\;X_{ij}+t_{ij}\;X_{ij}$$

(2) $$s.t.\underset{j\in N,j\neq i}{\sum }X_{ij}=1$$

(3) $$\underset{i\in N,i\neq j}{\sum }X_{ji}=1$$

(4) $$\underset{j\in N,j\neq 0\;}{\sum }X_{0j}=V\;\mathrm{and}\;\underset{i\in N,i\neq 0\;}{\sum }X_{i0}=V$$

(5) $$\underset{i\in N}{\sum }\underset{j\in N\left\{0\right\},i\neq j}{\sum }d_{i}\;{x_{ij}}^{n}\leq Q\forall n\in N$$

(6) $$\underset{i\in N}{\sum }\underset{j\in N\left\{0\right\},i\neq j}{\sum }t_{i}\;{x_{ij}}^{n}=T\;t_{i}\in T$$

(7) $$\underset{i\in N}{\sum }\underset{j\in N\left\{0\right\},i\neq j}{\sum }dis_{i}\;{x_{ij}}^{n}=Dis\;dis_{i}\in Dis$$

Where,

-
N denotes a group of hospitals, with $N=\left\{1,2,3,\ldots ,15\right\}$ .
-
$c_{ij}$ is cost of travel.
-
A denotes arcs.
-
V represents the total number of agents in the system.
-
Q is the vehicle capacity.
-
$d_{i}$ hospitals waste and deliver them to a disposal site.
-
$t_{ij}$ is a time of travel over arc $\left(i,j\right)\in A$ , T mean summation of total time $\left(t_{ij}\right)$ .
-
$dis_{ij}$ is a distance of travel over arc $\left(i,j\right)$ .
-
dis is a summation of total time $\left(dis_{ij}\right)\text{.}$

To compute this mathematical model, RL and DRL algorithms, such as Q-learning and DQN, are utilized. Additionally, each algorithm is adapted to address the vehicle routing problem, estimating not only the shortest route between agents but also allocating each shortest route to a designated number of agents based on their maximum capacity.

## 4 Proposed algorithm

Our closed capacity vehicle routing problem has been addressed using multiple mathematical learning approaches such as QL, DQN, a hybrid of DQN with the A* algorithm, and a hybrid of DQN with the fractional knapsack problem. The following subsections introduce each algorithm in the context of our problem.

### 4.1 Q learning approach

Q-learning is used to enable an interactive system to learn how to make optimal decisions in each environment to achieve specific goals. It is an offline temporal difference RL algorithm. A set of agents $V=\left\{1,2,\ldots ,15\right\}$ is considered, where each agent picks up waste from hospitals $h=\left\{1,2,\ldots ,15\right\}$ and delivers it to the disposal site (node 16) on a m×m grid. Q-learning is defined by a set of states, actions, and a Q-matrix. The states represent the various conditions or configurations the system can be in during the waste collection process. Actions refer to the specific choices available to the agent for movement at each state. The Q-matrix, or Q-table, stores the estimated values or rewards associated with taking specific actions in specific states. The different components of the model are detailed below in Supplementary Figure 1 (Kalakanti et al., 2019).

Supplementary Figure 1 presents the RL framework adopted for our specific case study. The formulated model is designed to determine the optimal shortest routes between hospitals while considering the waste collection capacity of the agents. A 17×17 grid was created where each cell contains a distance value based on recommendations. Initially, the values in the Q-matrix are set to zero. The Q-learning algorithm is used to iteratively improve these values through trial-and-error learning. The system updates the values interactively by assessing the disparities between current values and target values, which are computed using the Bellman equation (Nazib and Moh, 2021).


(8) $$Q\left(s_{t},a_{t}\right)\leftarrow Q\left(s_{t},a_{t}\right)+\alpha \left[r_{t+1}+\gamma \max_{a}Q\left(s_{t+1},a\right)-Q\left(s_{t},a_{t}\right)\right]$$

where $\alpha \in \left(0,1\right)$ is the learning rate.

It’s important to note that the Bellman equation has been adjusted to prioritize minimizing distance, time, and cost while finding the shortest routes between nodes. The original equation typically uses the max function, but our adaptation focuses on minimization instead. To align our problem with the Q-learning algorithm’s requirements, each arc $e_{ij}\in A$ is considered as 1eij , which involves adjustments to the adjacency matrix. However, managing the state space becomes increasingly complex in our model due to the presence of multiple agents and their concurrent positions on the grid. This complexity grows with larger numbers of vehicles and grid sizes, posing challenges for the Q-learning algorithm to derive optimal policies in larger problem instances.

#### 4.1.1 States

The set of states in our health care problem can be characterized as $S=\left\{v,h,d\;\right\}$ ; Were:

-
v represent Agent (vehicles)
-
h represents the hospital node
-
d represents a disposal site where waste is generated from hospitals.

moreover, for a given s∈S , $s_{i}$ describe the state dimension for $s_{i}=\left\{v_{i},h_{i},d_{i}\;\right\}$ ;Were:

-
$v_{i}$ constitutes the set of vehicle locations.
-
$h_{i}$ constitutes the set of hospital locations.
-
$d_{i}$ constitutes the set of disposal based on the location of hospitals.

In the first each agent is centralized in the depot zone in the first cell with index S=0 within the grid the variety of potential hospital locations is equal to the rest number of the grids from1⋯to15 . Demands of hospital waste accumulation take place within matrix cells that correspond to distinct hospital premises. Disposal sites can be found in specific location cells of the grid in addition to the hospital location inside the grid with index S=16 . The values in the grid represent the distances between hospitals, from each hospital to the depot, and from the hospitals to the disposal location. If there is no route between any two hospitals, the distance value is-1. When the agent reaches the disposal site, the maximum value in the grid (Reward), equal to 100, is assigned. Supplementary Figure 2 illustrates a random scenario of the locations for each hospital, depot, and disposal site inside a matrix. In this configuration, agents collect wastes from hospitals and then deposit them in the disposal site.

#### 4.1.2 Actions

Permission must be obtained for using copyrighted material from other sources (including the web). For every individual agent 𝑣∈𝑥, the set of available actions is 𝑋 = left, right up, down, pick−up, and .

It’s crucial to emphasize that the first four actions in our model determine the direction of movement within the grid (Laidlaw et al., 2023). When the agents’ location aligns with their load capacity, they will collect waste from hospitals. Once an agent reaches its maximum load capacity, it is required to deliver the waste to the specified disposal location. The primary objective of our model is to estimate the shortest route while maximizing the vehicle’s capacity and minimizing time, as illustrated in Supplementary Figure 3.

In Supplementary Figure 3, if a state is randomly selected, such as Hospital-1, the agent remains in the current state by choosing the shortest route between this hospital (current action) and the next. For example, if the agent selects Hospital-2 as the shortest route from Hospital-1, and let us assume the next action chosen is to the left of Hospital-1 according to the matrix containing the distances between the hospitals, then the next state is Hospital-2, towards which the agent moves.

#### 4.1.3 Reward

For every individual agent v∈x , a set of available actions is The primary aim of Q-learning is to guide the agent to the state with the maximum reward. Once the agent reaches this goal state, it stays there permanently. To determine the current action ( $a_{i}$ ) from the current state ( $s_{i}$ ), the agent follows the *ε*-greedy policy (Gilbert et al., 2023). This action is executed in the environment, resulting in feedback in the form of a reward ( $R_{i}$ ) and the next state ( $s_{i+1}$ ). The value inside the grid represents the distance between each hospital, between the hospital and the Directorate of Health Affairs location, and finally between hospitals and the disposal location. When the agent successfully reaches the goal state, it receives the maximum reward, which is set to 100. However, if the agent fails to reach the target state, it is penalized with a negative value. This penalty discourages the agent from failing. Incentivizing it to find alternative routes or strategies to reach the desired goal state. By adjusting the rewards and penalties, the agent can learn from its experiences and optimize its decision-making process to achieve higher success rates in reaching the goal.

#### 4.1.4 Enhancement

The plan involves implementing QL to address the CCVRP and determine the optimal route for each hospital. After that, capacity constraints are incorporated into the Q-learning approach. As part of this refinement, modifications are made to the QL algorithm to address capacity limitations, leading to the development of Capacity-constrained QL (CQL), which allows each vehicle to manage loads of up to 3 tons. The result of Q-learning is a separate greedy traversal of the shortest route for each hospital. To enhance this, the number of agents serving groups of hospitals is inserted based on their respective capacities. When applying this challenge, the results were unsatisfactory because the agent did not fully utilize its available capacity, and there were too many agents providing the service. Therefore, it was necessary to use algorithms that train the agents more effectively to provide useful outcomes for solving the problem. In the next section, the approach used will be DQN.

### 4.2 DQN approach

DQN is an advanced reinforcement learning algorithm that combines deep learning with Q-learning, a fundamental approach in reinforcement learning. Utilizing a deep neural network, DQN approximates the Q-function, which forecasts the anticipated future rewards based on actions taken within a given state. In the context of CVRP, DQN applies deep learning capabilities to tackle the intricate decision-making required for vehicle routing under capacity constraints, thereby providing improved and efficient solutions for route planning. In our problem scenario, DQN involves multiple agents (Majid et al., 2023). It experiences training across numerous time steps and episodes to optimize performance. The DQN model configuration includes two neural networks: The Q Network and the Target Network, as shown in Supplementary Figure 4.

Supplementary Figure 4 illustrates the DQN architecture. In this scenario, there is a 17×17 grid with the following inputs to the neural network.

- Current Location: 17 inputs (one hot encoded vector representing 15 hospitals, 1 central collection point, and 1 disposal center).
- Remaining Load Capacity: 1 input (single scalar value).
- Visitation Status: 15 inputs (binary vector for the visitation status of each hospital).
- Demands: 15 inputs (demands of each hospital).
- Distances: 17 inputs (distances from the current location to each of the 17 locations).
- The total number of inputs is 65.

The network has two hidden layers, with the first hidden layer consisting of 128 neurons and the second hidden layer consisting of 64 neurons. The activation function used for the hidden layers is (Rectified Linear Unit) $\mathrm{ReLU}\left(x\right)=\max\left(0,x\right)$ , while the activation function for the output layer is Linear $f\left(x\right)=\mathrm{ax}+b$ , as it outputs continuous Q-values. The architecture includes two neural networks: The Q Network and the Target Network. The Q Network is responsible for predicting the Q-values for each action given a specific state. It is updated frequently and learns the optimal policy by minimizing the difference between predicted Q-values and the target Q-values. The Target Network provides stable target Q-values during training. It is updated less frequently, such as every few thousand steps, with the weights of the Q Network. This helps stabilize the training process by providing consistent Q-value targets, preventing oscillations and divergence. The DQN formula is derived from the Bellman equation for Q-learning (Equation 7). In practice, DQN uses a neural network to approximate the Q-function. The formula for DQN is structured around Bellman’s equation (Moussaoui et al., 2023).


(9) $$Q\left(s_{t},a_{t}\right)=\mathrm{reward}\;\mathrm{if}\;\mathrm{the}\;\mathrm{new}\;\mathrm{state}\;\mathrm{is}\;\mathrm{the}\;\mathrm{terminal}\;\mathrm{state}\ldots .\mathrm{else}$$

(10) $$Q\left(s_{t},a_{t},\theta \right)\leftarrow r+\alpha \;\gamma \max_{a}Q\left(s_{t+1},a_{t+1},\theta^{\prime }\right)$$

where r is the reward, γ is the discount factor, α is the learning rate, Q Network weights with parameters θ and target network weights with parameters $\theta^{\prime }$ . Effective neural network training requires a well-defined loss (or cost) function. In the context of DQN, this function is formulated as the squared difference between the two terms of the Bellman equation, commonly referred to as the DQN loss function.


(11) $$\mathrm{Loss}={\left(r+\gamma \max_{a}Q\left(s_{t+1},a_{t+1},\theta^{\prime }\right)-Q\left(s_{t},a_{t},\theta \right)\right)}^{2}$$

As the number of hospitals increases, employing a DQN introduces several challenges, such as high-dimensional state and action spaces, scalability issues, training instability, difficulty in balancing exploration and exploitation, insufficient state representation, significant computational demands, and the risk of suboptimal solutions. Combining DQN with the A* algorithm offers an effective strategy to overcome these challenges.

### 4.3 Hybrid A* with DQN approach (A*DQN)

A* is a widely used pathfinding algorithm in computer science and artificial intelligence that efficiently finds a route from a starting node to a goal node within a graph or grid. A* employs a heuristic to guide its search, evaluating nodes based on their cumulative cost from the start node (referred to as the “g-value”) and the estimated cost to reach the goal node (referred to as the “h-value”). It prioritizes nodes for exploration by combining these values to identify the most optimal path. During each iteration of its main loop, A* selects the path to extend by considering both the cost of the current path and the estimated additional cost to reach the goal, specifically choosing the path that minimizes the total cost, as defined by the following equation (Yan, 2023).


(12) $$f\left(n\right)=g\left(n\right)+h\left(n\right)$$

Here, n represents the next node on the path, $g\left(n\right)$ denotes the cost of the path from the start node to n , and $h\left(n\right)$ is a heuristic function that estimates the cost of the most efficient path from n to the goal. These values are extracted from the 17×17 distance matrix between hospitals, previously employed by Q-learning and DQN. The A* algorithm was applied to the problem and then hybridized with DQN to enhance the solutions. This integration of the two algorithms is shown in Supplementary Figure 5.

Supplementary Figure 5 shows that, given the specified environment, including hospital locations, their numbers, and the distances between them, the A* algorithm was initially used alone to generate and present the results. These results were then refined using DQN. To integrate the A* algorithm with DQN for solving the CVRP problem, A* is first applied to generate initial routes between hospitals, the central collection point, and the disposal center. A* provides a preliminary solution based on distance and waste load. Then, DQN is used to optimize these routes through continuous learning. DQN utilizes a Q Network to estimate the value of different actions, while the Target Network helps stabilize the training process. Combining A* for initial route planning with DQN for dynamic optimization enhances solution efficiency, improves resource utilization, and reduces transportation costs. This method leverages the strengths of both algorithms: A* provides a robust starting point, and DQN offers ongoing improvements based on accumulated experience. Given the increase in the number of hospitals operating on a larger scale, meaning that this system works with a group of provinces together, the quality of computing solutions for this algorithm becomes more complex as it requires greater computational resources due to dealing with a larger number of potential routes directly. Therefore, it is better to group nearby hospitals into one zone to make it easier to handle a larger number. This will be explained in the next section.

### 4.4 K-means cluster with DQN approach (CDQN)

Clustering hospitals for CVRP involves grouping them based on proximity, which simplifies the routing process and reduces computational complexity. This approach allows each cluster to be treated as an independent CVRP, making it easier to identify optimal routes. By minimizing the number of potential routes evaluated, clustering improves efficiency and facilitates parallel processing, significantly accelerating the solution process. Moreover, clustering enhances scalability as the number of hospitals increases, preventing excessive complexity. It also enables localized optimization, allowing specific characteristics of each cluster to inform better route planning (Sinaga and Yang, 2020). Overall, clustering transforms the CVRP into manageable sub-problems, leading to more effective solutions. K-Means was utilized to perform the clustering process, as illustrated in Supplementary Figure 6.

Supplementary Figure 6 illustrates the K-Means clustering applied to the CVRP. The K-Means algorithm is a widely used method for partitioning a set of points into a predetermined number of clusters (K) based on proximity. Its objective is to minimize the distance between points(hospitals) within each cluster while maximizing the distance between different clusters. The process starts with randomly selecting K initial cluster centers, assigning each point to the nearest center, and iteratively updating the centers until they stabilize. Steps to Execute K-Means (Morissette and Chartier, 2013):

- Step 1: Initialize Cluster Centers: Randomly select K initial centroids from the dataset.

(13) $$C=\left\{c_{1},c_{2},c_{3},\ldots \ldots c_{k}\right\}$$

- Step 2: Assign Points to Clusters: Assign each data point $h_{i}$ to the closest centroid $c_{k}$ .

(14) $$\mathrm{Assign}\left(h_{i}\right)=\arg\min_{k}||h_{i}-c_{k}||{}^{2}$$

- Step 3: Update Cluster Centers: After assigning all points, determine the new centroid for each cluster $c_{k}$ by calculating the average of all points in that cluster

(15) $$c_{k}=\frac{1}{\left|S_{k}\right|}\;\underset{h_{i}\in S_{k}}{\sum }h_{i}$$

- Step 4: Check for Convergence: Repeat steps 2 and 3 until the centroids change only slightly or the assignments remain unchanged. A common criterion for convergence is

(16) $$||c_{k}^{\mathrm{new}}-c_{k}^{\mathrm{old}}||<\in$$

- where ∈ epsilonϵ is a small threshold.
- Step 5: Output the Final Clusters: after convergence is achieved, provide the final cluster assignments and centroids.

After applying the K-means algorithm to divide the specified hospitals on the Google map, the results revealed the number of hospitals and their names within each zone. This is where the DQN algorithm, as previously discussed, is utilized to find the shortest path between the hospitals within each zone individually. When applying K-means clustering with DQN, it was found that while it ensures maximum capacity for some agents, it does not do so for all. Additionally, there are constraints arising from this integration when the number of hospitals is larger at the provincial level, which may lead to suboptimal partitions from K-means that affect the final solution. Therefore, it was necessary to integrate certain algorithms with DQN to address these issues and ensure that each agent reaches its maximum capacity, such as the Fractional Knapsack Problem.

### 4.5 Fractional knapsack problem with DQN APPROACH (FKPDQN)

The Fractional Knapsack Problem (FKP) is an optimization challenge that aims to maximize the total value of items that can be placed in a knapsack without exceeding a specified weight limit. Unlike the 0/1 Knapsack Problem, where only whole items can be selected, the FKP allows for the inclusion of fractional quantities of items. Each item is defined by its weight and value, and the objective is to select items in such a way that the total value is maximized while staying within the weight capacity of the knapsack.

To solve the Capacitated Vehicle Routing Problem (CVRP) using dynamic programming alongside the Fractional Knapsack Problem, we break down the larger problem into smaller, manageable subproblems. By combining dynamic programming with the Fractional Knapsack Problem and using Deep Q Networks (DQN), we can find effective solutions to complex challenges. Dynamic programming assists in decomposing the problem, while DQN is employed to formulate decision-making policies in uncertain environments like CVRP.

By leveraging results from dynamic programming to estimate optimal values, the performance of DQN is enhanced through improved state evaluations. This hybrid approach enhances the decision-making capability of the model by integrating optimal solutions with experiential learning, as illustrated in Supplementary Figure 7.

In Supplementary Figure 7, the solution to the Fractional Knapsack Problem in a waste management environment is illustrated. This solution aims to achieve the shortest path between hospitals to collect waste. In this environment, each hospital collects hazardous waste in a set of bags, each weighing 30 kg. When the agent visits the hospital, it collects these bags (Windfeld and Brooks, 2015). The concept of the Fractional Knapsack is applied, meaning it is not necessary to collect all the waste bags from a single hospital in one visit. This is because the agent’s capacity may be full, leaving no room for the remaining bags. As a result, the hospital may be visited multiple times by different agents. For instance, in the illustrated figure, the agent collected all the bags from Hospitals 1 and 2. However, when visiting Hospital 3, it collected two bags and left one bag to be picked up by another agent, as multiple agents are used. If the 0/1 Knapsack approach were applied, the agent would leave Hospital 3 without collecting any bags if the remaining capacity was insufficient for all the hospital’s waste, thus wasting some of the agent’s capacity. Below are the steps to implement a waste management solution using the Fractional Knapsack algorithm (Jain, n.d.):

- Step 1: Define the Problem: Clarify both of the following: items(n) =15 hospitals,one depot,and one disposal site
- Each item has a weight (waste)={w_i_}.
- Each item has a value (profit)(v)={1/(cost for transporting wastes)_i_}.
- A knapsack with Total capacity W =3 Tons.
- The objective is to maximize the total value V in the knapsack without exceeding its capacity W .
- Step 2: Initialize a DP Table: Create a 2D DP table where $d\left[i\right]\left[w\right]$ .
- i represent several items, initialize from 0 to i.
- w represents the maximum allowable capacity of
- agents, initialized from 0 to W.
- Step 3: Populate the DP Table: the dimension of the table equals (number of items +1) × (capacity of the knapsack +1). The first row of the DP table is filled with zeros, representing the base case where no items are included, which results in a maximum value of zero, regardless of the knapsack’s capacity. The remaining values in the table are filled using the following equation.

(17) $$d\left(i,w\right)=\max\left(d\left(i-1,w\right),d\left(i-1,w-w_{i}\right)+v_{i}\right)$$

- Step 4: Steps to Extract the Result: To extract the result after filling the DP table.
- Identify the Final Cell: The maximum value that can be obtained with the knapsack of capacity will be stored in $d\left[i\right]\left[w\right]$ .
- Backtrack to Find the Items Included:

1. Start from $d\left[i\right]\left[w\right]$ .
2. Check if the value came from the inclusion of the current item or from the previous item without including the current item.
3. If the value came from the inclusion of the current item, add this item (or the fractional part of it) to the solution list.
4. Adjust the remaining capacity of the knapsack accordingly.
5. Move to the next item and repeat until you reach $d\left[0\right]\left[0\right]$ .

After following all the steps, the shortest route for each agent is determined using dynamic programming for the Fractional Knapsack Problem. This approach ensures that all agents can fully utilize their 3-ton capacity without leaving any unused space. By dividing the waste among all hospitals and determining the shortest path between them, the solution from the Fractional Knapsack Problem becomes the input for the DQN algorithm in the neural network. This output is then trained and improved by comparing it with the DQN output to obtain optimal solutions, as previously explained in detail for DQN.

All agents tasked with finding the shortest route between hospitals for collecting and transporting waste to the disposal center have a carrying capacity of 3 tons. These vehicles require fuel, either 92-octane gasoline or diesel. In this study, 92-octane gasoline is used. The average fuel efficiency for transport vehicles up to 5 tons is 6.5 km/liter (Muñoz et al., 2022). The cost for each agent will be calculated based on the distance covered, starting from the moment the vehicle leaves the depot, traveling along the shortest route between hospitals, reaching the disposal center, and returning to the depot. Finally, a comparison will be made between the average fuel cost for these agents and the cost of operating battery-powered agents (Kaleg et al., 2015).

## 5 Study results

The experimental case study presented in this paper applied the proposed artificial intelligence techniques described earlier to address the CVRP problem. The implementation was carried out in Python on a Windows 10 (64-bit) system featuring a 3.10 GHz CPU, 16 GB of RAM, and a 512 GB SSD. The results provide insights into the number of agents used, the shortest routes assigned to each agent, and the load capacity transported by each agent along their respective routes.

### 5.1 CQL result

Upon training our approaches to address the identified issue during the parameter tuning process, such as the learning rate *α* and discounting factor *γ*, the optimal training selection for each parameter was found to be α=.9 and γ=.8 . Q-learning algorithm is utilized to optimize our problem of finding the shortest route between hospitals while considering the waste amount from each hospital and the maximum capacity of the agent, set at 3 tons. The output includes a greedy traversal route designed for each hospital. We modified the Q-learning implementation to incorporate the constraints of the Capacitated Vehicle Routing Problem (CVRP). Our goal is to establish connections between the different routes assigned to each hospital, using multiple agents for service. Additionally, we need to set a constraint: if the medical waste generated by a single hospital reaches approximately 3 tons, the assigned agent will serve that hospital exclusively and then proceed directly to the waste disposal center. Specifically, node 15, which represents the Liver Institute, generates about 3 tons of waste with each visit. As a result, this hospital has been assigned a dedicated agent, as each agent has a capacity of 3 tons. These agents begin their trips from a collection center, visit hospitals to collect waste, transport it to a designated disposal site, and then return to the collection center, as shown in Supplementary Table 1.

In Supplementary Table 1, the first column lists the number of vehicles (Agents No.) employed by CQL to address the waste collection issue. The second column outlines each vehicle’s route, starting from the depot, passing through nearby hospitals, and reaching the waste disposal site during the first visit of the week. The third column displays the total waste capacity collected along each vehicle’s route during these hospital visits. The fourth column details the vehicle routes for the second visit within the same week, which occurs 3 days after the first. The fifth column indicates the waste capacity generated by the hospitals during the second visit of the week.

This table identifies the shortest routes for all hospitals by deploying multiple agents, each according to its maximum capacity. For instance, the first column indicates that a total of seven agents were assigned, as determined by CQL, to manage waste collection across all hospitals. The second column presents the shortest route for each agent. For instance, Agent-1 ( $A_{1}$ ) follows the route (C → 15 → D → C), starting at depot $\left(C\right)$ , moving to node 15, and continuing to the next node until the agent’s capacity reaches 3 tons. Once this capacity is reached, the agent proceeds to the disposal site $\left(D\right)$ to offload the waste and returns it to the depot, adhering to the closed CVRP model. This process occurs during the agent’s first visit of the week.

Columns 3 and 5 display the route and vehicle capacity during the second visit in the same week. Agent $A_{1}$ serves only the Liver Institute node, as it reaches its maximum capacity after collecting waste. This node is visited three times per week due to the accumulation of waste, totaling nearly 3 tons. For instance, $A_{2}$ selects the shortest route, which involves visiting Quwisna General Hospital first, then Qasr Hospital, before heading to the disposal site to dispose of the waste and returning it to the collection center. On the $1^{st}$ visit, the agent handles 2.379 tons, and on the $2^{nd}$ visit, the total capacity is 2.46 tons over the week. It is noted that the agent’s route during the $1^{st}$ visit is identical to that of the $2^{nd}$ visit, but the capacity varies. This variation can be attributed to the changing amounts of waste generated by the hospitals each time.

Ultimately, it was observed that the value of each agent did not reach its maximum capacity. Therefore, a more precise training and testing process is required to fully utilize the maximum capacity of each agent. Hence, DQN is selected for this purpose.

### 5.2 DQN result

Upon training our approaches to address the identified issue After applying the DQN approach, the optimal tuning parameters for each variable are as follows: α=.001 , $\gamma =.95,\varepsilon \;\left(\mathrm{Exploration rate}\right)$ starts at 1.0 and gradually decaying to a minimum value of 0.01 . The multi-agent system utilizes exploration and exploitation during training to determine the optimal shortest route for each agent, serving the nearest hospital, as shown in Supplementary Table 2.

Supplementary Table 2 presents the optimal solution for each agent in serving a group of hospitals. $A_{1}$ exclusively served node 15, as discussed previously. The remaining agents served the rest of the group of hospitals. For instance, $A_{2}$ visited node 1, then node 2, followed by node 9, and finally, node 6, disposing of wastes at the disposal site and returning to the collection center with 2.981 tons during the first visit and 2.997 tons during the second visit, depending on the extracted waste amount. The total number of agents used to serve our problem during the first and second visits is five. It’s important to note that the DQN-trained network aims to find the shortest route while considering the maximum capacity, which is close to 3 for this agent compared to the Q-learning approach. However, not all agents consistently achieve their maximum capacity. Combining A-star with DQN will contribute significantly to enhancing these outcomes.

### 5.3 A* result

Initially, A* was implemented exclusively to find the shortest path by assigning the values of g(n) and h(n) between each node and between each node with the collection center and the disposal site, represented as a matrix. This resulted in the optimal path for each agent, as shown in Supplementary Table 3.

Supplementary Table 3 illustrates the optimal path for each agent. During the week’s first visit, all hospitals were served by four agents, but in the second visit, five agents were used. Hospital 3, which was initially served by agent $A_{2}$ , was served by agent $A_{5}$ on the second visit. This change was due to the amount of waste extracted during the second visit exceeding the maximum capacity of agent $A_{2}$ , requiring the assistance of another agent. Some agents’ capacities are close to 3 tons, indicating that the A* approach effectively addressed our problem. This efficiency suggests that integrating A* with DQN could further enhance the network, enabling the Q-network to overcome near-optimal results when using DQN alone and ultimately determine the optimal solution for each agent through hybridization as shown in Supplementary Table 4.

Supplementary Table 4 depicts the integration of A* with DQN, which improves the outcomes of the DQN approach. In this hybridization, agents’ capacities are closer to the optimal 3 tons, unlike when using DQN alone. In the upcoming section, a clustering approach will be introduced to categorize hospitals before the implementation of the DQN network, aiming to evaluate enhancements in the outcomes.

### 5.4 CDQN result

After applying the K-means clustering approach, which divided all hospitals on Google Maps into three zones with the nearest nodes grouped, the Directorate of Health Affairs node was chosen as the centroid based on the points of other hospitals. The shortest route within each zone was then determined using the DQN approach as shown in Supplementary Table 5.

Supplementary Table 5 illustrates how the K-means approach divides all hospitals into three zones: the first zone includes nodes 2, 5, 11, 12, and 15; the second zone includes nodes 3, 6, 7, 8, 10, and 13; and the third zone includes nodes 1, 4, 9, and 14. During the first visit, node 15 contained nearly 3 tons of waste, so the agent served only this hospital. The second agent served the remaining hospitals in this zone using the DQN approach to find the shortest route between them. The third agent served the second zone, also using the DQN approach to consider the shortest route between hospitals. The fourth agent served the third zone. However, during the second visit, the second agent could not serve all hospitals in its zone because its capacity exceeded 3 tons, so it only served nodes 2, 5, and 11. Similarly, the fourth agent did not serve all nodes in zone three; it served only nodes 4, 1, and 9. Consequently, nodes 12 and 14 were not served, and a fifth agent was required to serve these two hospitals while respecting the shortest route. Finally, it was observed that all the approaches aimed to achieve the shortest route between hospitals while maximizing the capacity of each agent. However, if there is a small amount of remaining capacity, such as one-eighth of a ton, the vehicle would go to the disposal site to offload it because there is no hospital with just one-eighth of a ton of waste. Therefore, it is suggested that the waste in the hospitals be divided to utilize the remaining capacity of the agents. In other words, the waste in the hospitals should be split so that part of it fills the remaining capacity of one agent, and the other part is served by another agent.

### 5.5 FKPDQN result

Every hospital produces a certain amount of waste. According to the fractional knapsack approach, it is important to know how many bags of waste each hospital produces out of the total amount of waste it generates. This approach relies on collecting part or all of the waste from a hospital based on the available capacity of each agent during their visit to any hospital. Since the capacity of a single waste bag in any hospital is 30 kg, a hospital may need to be visited once or multiple times depending on the available load capacity of the agent. Here is an explanation of the shortest path between hospitals using this algorithm, as shown in Supplementary Table 6.

Supplementary Table 6 shows that four agents were utilized during both the first and second visits. Each agent can carry a maximum capacity of 3 tons, but the fourth agent did not reach this capacity because all nodes were serviced and no additional nodes remained. Node 4 was visited by two different agents. Initially, $A_{2}$ serviced node 4 and loaded 2 bags, along with bags from other nodes along the same path. Subsequently, $A_{3}$ loaded the remaining 34 bags at node 4, following a different path and servicing different nodes compared to $A_{2}$ . Similarly, node 2 was serviced by $A_{3}$ and $A_{4}$ . $A_{3}$ serviced nodes 4, 5, 8, and 2, taking only 21 bags from node 2. The remaining 29 bags from node 2 were serviced by $A_{4}$ , along with its assigned path. Node 15 was served three times a week by agent $A_{1}$ , with a total load of 100 bags each time.

## 6 Discussion

In general, an EV will submit a charging request when the strategic plans must be developed to determine agents who will visit hospitals periodically and efficiently. By optimizing the number of agents visiting hospitals, economic savings can be achieved in terms of fuel, agent wages, vehicle maintenance, as well as the time, distance, and cost associated with waste transportation.

The proposed approach integrates advanced reinforcement learning techniques, including Q-learning, DQN, and hybrid methods such as DQN combined with fractional knapsack and A* algorithms, to address the complexities of medical waste management. A comparison of various algorithms highlighted their effectiveness in optimizing resource utilization. For instance, using CQL required 7 agents to cover all hospitals with a visitation rate of twice per week, while DQN reduced this number to 5 agents for the same rate. A* initially assigned 4 agents for the first visit but needed 5 for the second visit. Both the combination of DQN and A* and Cluster DQN required 4 agents for the first visit and 5 for the second. FKPDQN outperformed others by achieving a consistent outcome of requiring only 4 agents for both visits. Although the number of agents assigned by algorithms like A*, DQN, A*DQN, and CDQN was similar, with each agent’s capacity approaching the total limit of 3 tons, none of these algorithms reached full capacity utilization. In contrast, FKPDQN proved to be the most effective, not only reducing the fleet size to 4 agents but also ensuring that each agent achieved the maximum capacity of 3 tons.

These results demonstrate the practical implications of the approach, including reduced fleet size, optimized capacity utilization, and minimized operational costs. By assigning existing agents with the remaining capacity to service additional hospitals, the approach further reduces the distance traveled and minimizes return trips, resulting in significant savings in fuel, time, and maintenance costs. The scalability of techniques like clustering (e.g., K-means) and hybrid models ensures efficient management of large-scale hospital networks. Moreover, the framework promotes sustainability by encouraging the use of electric vehicles, reducing emissions, and lowering fuel dependency, making it environmentally friendly.

Ultimately, the preference for algorithms requiring fewer agents aligns with the goals of reducing fuel consumption, costs, and environmental impact. The ability of FKPDQN to utilize all agents to their full capacity while minimizing the number of required agents positions it as the optimal choice in medical waste management. This balance between operational efficiency and environmental sustainability underscores the practical and scalable benefits of the proposed approaches. The distance and time covered by each algorithm will be presented subsequently in Supplementary Figures 8, 9.

Supplementary Figures 8, 9 show the total distance covered during two visits in a week using six different approaches established in our problem. The distance for $A_{1}$ remains the same across all approaches because this agent exclusively serves node 15, which generates 3 tons of waste, matching the agent’s capacity. The distances traveled by the other agents differ across the various approaches, depending on the hospitals they visit. The total distance traveled by multi-agents (from A1 to A7) between hospitals during the first visit is as follows: 622.34 km for CQL, 487.6 km for A*, 609.2 km for DQN, 489.33 km for A* DQN, 492.33 km for CDQN, and 580.2 km for FKPDQN. In the second visit within the same week, CQL’s distance remains unchanged at 622.34 km. For the other methods, the distances are: A* covers 529.6 km, DQN remains at 609.2 km, A* DQN covers 588.3 km, CDQN covers 599.43 km, and FKPDQN remains at 580.2 km. It is observed that the longest distance traveled by multi-agents was achieved using FKPDQN. This is because FKPDQN aimed to optimize the maximum value for each agent; it does not head to the depot if there is still available capacity but instead looks for the nearest hospital to fill the remaining capacity, even if it means taking some waste from that hospital. In contrast, the other approaches also sought the shortest route while considering capacity, but they operated on a 0/1 loading waste system from hospitals. Therefore, FKPDQN achieved longer distances because it visited more hospitals to fully utilize the available 3-ton capacity. When the number of multi-agents used is reduced, it improves the quality of the algorithm because saving an agent means saving the distance they would have traveled. The distances that the removed agent would have covered are distributed among the remaining agents who are already serving the hospitals until they reach their total capacity of 3 tons. If the number of agents used to serve the hospitals is ranked while achieving maximum capacity, FKPDQN performs the best among the other approaches used here. Supplementary Figures 10, 11 present the time metrics for each approach.

Supplementary Figures 10, 11 show the time consumed during two weekly visits. In each approach that was implemented, every agent travels a specific distance until completing its route, and this distance corresponds to the time for the agent. The total time consumed by multi-agents (from A1 to A7) between hospitals during the first visit is as follows: 1006 min for CQL, 771 min for A*, 912 min for DQN, 770 min for A* DQN, 772 min for CDQN, and 913 min for FKPDQN. During the second visit within the same week, CQL’s time remains at 1006 min. For the other approaches, the times are: A* takes 841 min, DQN remains at 912 min, A* DQN takes 949 min, CDQN takes 940 min, and FKPDQN stays at 913 min. Ranking the time consumed according to the distance calculated for each approach shows that FKPDQN is the best and CQL is the worst. Each waste transfer from the hospital to the disposal site by agents incurs a fee as shown in Supplementary Figures 12, 13.

Supplementary Figures 12, 13 illustrate the fees incurred for transporting waste from each hospital to the disposal site based on the amount of waste from each hospital. The average cost of transporting one ton of waste from hospitals is half a dollar. To calculate the waste transportation cost for any hospital, multiply the amount of waste in tons by half a dollar. $A_{1}$ incurs the same fee across different approaches because it consistently serves node 15, while other agents experience varying costs across different approaches. The total waste fees for multiple agents (A1 to A7) between hospitals during the first visit are uniformly $6.40 across all methods: CQL, A*, DQN, A* DQN, CDQN, and FKPDQN. On the second visit within the same week, the fee increases to $6.51 for all methods, including CQL, A*, DQN, A* DQN, CDQN, and FKPDQN. The cost of waste transportation from hospitals remains constant across all algorithms, despite differences in routes and the number of agents used. This consistency is because each algorithm serves the same number of hospitals (15) with the same amount of waste, resulting in the same final transportation cost. Additionally, to operate effectively, agents require fuel to complete their journeys, as shown in Supplementary Figures 14, 15.

Supplementary Figures 14, 15 illustrate the transportation cost for each agent. It is essential to determine the fuel consumption for each agent, given that each agent has a capacity of 3 tons and the average distance covered per liter of fuel is 8 km/liter. This is achieved by calculating the distance each agent travels during their journey, dividing it by the fuel consumption per kilometer, and then multiplying the result by the specified fuel cost in Egyptian pounds. This allows for determining the actual fuel cost for each agent based on the distance traveled. All values are converted into dollars to comply with international standards. If these agents are fueled with 92 octane gasoline, which costs 12.5 Egyptian pounds per liter, the total fuel cost for each agent based on the total distance traveled is as follows: $20.39 for CQL, $15.97 for A*, $19.96 for DQN, $16.03 for A* DQN, $16.13 for CDQN, and $19.01 for FKPDQN during the first visit to the group of hospitals. During the second visit within the same week, the costs are: $20.39 for CQL, $17.35 for A*, $19.96 for DQN, $19.27 for A* DQN, $19.67 for CDQN and $19.01 for FKPDQN. The total fuel cost for agents using the CQL and FKPDQN algorithms remains the same during both the first and second visits of the week, as each agent follows the same route for waste collection, leading to identical distances traveled and, consequently, identical costs.

Naturally, reducing the number of agents results in savings in their usage, which translates to reduced fuel consumption and maintenance costs, all achieved with the FKPDQN algorithm. If these agents are switched from fuel to electric power, the economic impact is illustrated in Supplementary Figures 16, 17.

Supplementary Figures 16, 17 show the battery costs for each agent across different methods. Given the continuous rise in fuel prices in Egypt and the resulting harmful emissions, it is crucial to find solutions to these issues by converting fuel-powered agents to battery-powered ones. Charging these agents requires connecting them to a fast charger at charging stations, where a direct payment system is used. Battery-powered agents are more efficient and require less maintenance compared to those running on fuel. To calculate the cost for battery-powered agents, it is important to know that the average cost of electricity per kilowatt-hour is 1 Egyptian pound, and the battery efficiency allows for a range of 5 kilometers per kilowatt-hour for 3-ton agents. The distance traveled by the agent during their journey is also considered, and all values are converted into dollars for easier reading and international classification.

The battery costs for multiple agents covering different distances in each approach are as follows: $2.61 for the CQL algorithm, $2.04 for the A* algorithm, $2.55 for the DQN algorithm, $2.05 for the A* DQN algorithm, $2.06 for the CDQN algorithm, and $2.43 for the FKPDQN algorithm during the first visit to the group of hospitals. During the second visit within the same week, the costs are: $2.61 for the CQL algorithm, $2.22 for the A* algorithm, $2.55 for the DQN algorithm, $2.47 for the A* DQN algorithm, $2.51 for the CDQN algorithm, and $2.43 for the FKPDQN algorithm.

When comparing the cost of fuel to using batteries, it is clear that battery costs are significantly lower. Therefore, battery-powered agents are preferred for CVRP problems. This preference is not only due to the higher fuel costs compared to batteries but also because battery use reduces environmental pollution from fuel emissions and decreases the need for regular maintenance of agents. The worst-case scenario for waste collection was studied, which occurred during the second visit of the week. This was considered the worst case because the amount of waste generated by each hospital was larger than during the first visit. When evaluating the quality metrics across different algorithms, it was observed that the performance metrics were measured based on several key factors: the number of vehicles used, the utilization of vehicle capacity, distance traveled, time taken, fuel cost, and battery cost.

Supplementary Table 7 presents a comparative analysis in percentages, based on the reference values, to assess the performance of each algorithm. Supplementary Table 7 shows the Reference (Optimal) Values and the Percentage differences between the algorithms used. The FKPDQN algorithm is the best for minimizing the number of vehicles required, as it uses only 4 vehicles, achieving 0% loss compared to other algorithms. Additionally, it achieves 100% vehicle capacity utilization. Although it results in a higher distance and time compared to the algorithm with the lowest reference values, this is because it visits more hospitals to maximize vehicle capacity, thereby reducing the number of vehicles needed. Ultimately, the primary goal or key metric is to minimize the number of vehicles and fully utilize their capacity. So, the FKPDQN algorithm reduces the number of agents needed, leading to effective savings in cost, distance, and time.

## 7 Conclusion

The study, while demonstrating promising results, has certain limitations that require further elaboration. One of the main challenges is algorithmic scalability, as hybrid algorithms might encounter computational complexity when applied to larger datasets or multi-depot scenarios. Additionally, real-world integration poses practical implementation challenges, such as dynamic changes in hospital waste generation and fluctuating traffic conditions, which were not fully addressed in this study. Another limitation is the potential inefficiency of K-means clustering, which may lead to suboptimal groupings, especially in large provincial networks. Lastly, while the results are based on a specific case study, their broader applicability to other regions or contexts needs further validation to ensure their generalization.

In this paper, flexible reinforcement learning techniques were combined with optimization methods to train multiple agents for solving the CVRP in waste management. Several effective approaches were applied to address this problem, including CQL, A* with DQN, clustering with DQN, and FKPDQN. The most effective algorithm was FKPDQN, which integrates the fractional knapsack problem with DQN. This hybrid algorithm reduces the number of agents required to find the shortest path for collecting medical waste between hospitals and utilizes the maximum capacity of each agent, which is up to 3 tons, ensuring full utilization of available capacity without any wastage, compared to other methodologies used in this study.

The ranking of these approaches in terms of effectiveness is as follows: FKPDQN, followed by CDQN, A* DQN, A*, DQN, and finally CQL. Given that hospitals handle waste by dividing it into 30 kg bags, using the FKPDQN algorithm, which allows for collecting some or all of the waste according to the available agent capacity, is more effective than using the 0/1 knapsack problem that requires either taking all the waste or leaving it entirely, as seen with other approaches.

The options CDQN and A* DQN are nearly equal in terms of the following criteria: the number of agents used, the proximity of each agent’s capacity to 3 tons, and the reduction in distance and time per agent. However, CDQN has an advantage due to its ability to handle large hospital networks more effectively. In such large-scale scenarios, CDQN can implement a shared cluster agent, which reduces the number of agents needed and lowers both cost and time. On the other hand, A* and DQN compete similarly in terms of the factors contributing to algorithm quality. However, A* is more precise and faster in execution for small hospital networks because it functions as a heuristic search algorithm. For large hospital networks, DQN is preferred as it trains the agent extensively to achieve an optimal solution rather than a near-optimal one, which is the case with A* when applied to a larger number of hospitals.

The agents start their journey from a single depot, the “Directorate of Health Affairs,” where they search for the shortest path to collect waste from hospitals. They then fill their agent capacity and head to the only disposal site, “Kafr Dawood Al-Sadat,” to dispose of the waste. FKPDQN used 4 agents to find the shortest path and collect waste from 15 hospitals located in different areas on the map, covering a total distance of 580.2 km in 913 min, with a fuel cost of $19.01 for 92 octane gasoline. If the agents were operating on batteries, the savings would amount to $2.43.

Future work includes applying CVRP with a time window for hospital waste management. In addition to the previously mentioned DQN, other advanced deep reinforcement learning (DRL) algorithms, such as Proximal Policy Optimization (PPO), Twin Delayed Deep Deterministic Policy Gradient (TD3), and A3C (Asynchronous Advantage Actor-Critic), will be explored to improve decision-making and the overall optimization of routes. Additionally, performance could be further improved by using a multi-depot algorithm to achieve shorter routes and lower costs, and Open CVRP could be used to reduce the distance traveled. Moreover, to expand the applicability of the model, future research will involve integrating data from different provinces, enabling the use of shared cluster agents. These agents would optimize the routing by estimating the shortest routes between hospitals across regions, creating a more scalable and adaptable system for waste management. This research can provide solutions not only for hospital waste management within a single area but also across multiple regions, helping to further reduce costs and improve efficiency in large-scale urban environments.

## Data Availability

The original contributions presented in the study are included in the article/Supplementary material, further inquiries can be directed to the corresponding author.
