# Supplementary material for: Enhanced vehicle routing for medical waste management via hybrid deep reinforcement learning and optimization algorithms
Source: Front Artif Intell. 2025 Feb 12;8:1496653. doi: 10.3389/frai.2025.1496653 (PMC11861366; doi:10.3389/frai.2025.1496653)
Supplement: Supplementary file 1 [file Data_Sheet_1.pdf]

Supplementary Material

1 Supplementary Tables

The Tables related to the issue were collected separately and are used to support and analyze the data presented in the study

TABLE 1 Shortest route for each vehicle using CQL

No.	Vehicle Route in the week	Cap	Vehicle Route in the week	Cap
A_1	C → 15 → D → C	3	C → 15 → D → C	3
A_2	C → 1 → 2 → D → C	2.376	C → 1 → 2 → D → C	2.46
A_3	C → 3 → 7 → 8 → D → C	1.744	C → 3 → 7 → 8 → D → C	1.717
A_4	C → 4 → 9 → 10 → D → C	1.646	C → 4 → 9 → 10 → D → C	1.766
A_5	C → 5 → 6 → D → C	0.875	C → 5 → 6 → D → C	0.902
A_6	C → 11 → 12 → 13 → D → C	1.496	C → 11 → 12 → 13 → D → C	1.358
A_7	C → 14 → D → C	0.582	C → 14 → D → C	0.657

TABLE 2 Shortest route for each vehicle using DQN

No.	Vehicle Route in the week	Cap	Vehicle Route in the week	Cap
A_1	C → 15 → D → C	3	C → 15 → D → C	3
A_2	C→ 1 → 2 → 9 → 6 → D → C	2.981	C→ 1 → 2 → 9 → 6 → D → C	2.997
A_3	C → 4 → 8 → D → C	1.837	C → 4 → 8 → D → C	1.885
A_4	C → 10 → 13 → 11 → 12 → D → C	1.705	C → 10 → 13 → 11 → 12 → D → C	1.672
A_5	C → 5 → 3 → 7 → 14 → D → C	2.169	C → 5 → 3 → 7 → 14 → D → C	2.306

TABLE 3 Shortest route for each vehicle using A*

**No.**	**Vehicle Route in the week**	**Cap**	**Vehicle Route in the week**	**Cap**
A_1	C → 15 → D → C	3	C → 15 → D → C	3
A_2	C→ 1→ 2→ 6 → 3 → D → C	2.972	C → 1→ 2→ 6 → D → C	2.651
A_3	C → 4 → 9 → 10 → 5 → 7 → D → C	2.908	C → 4 → 9 → 10 → 5 → 7 → D → C	2.988
A_4	C → 13 → 11 → 12 → 8 → 14 → D → C	2.812	C → 13 → 11 → 12 → 8 → 14 → D → C	2.794
A_5	------------------	0	C → 3 → D → C	0.427

TABLE 4 Shortest route for each vehicle using A* with DQN

**No.**	**Vehicle Route in the week**	**Cap**	**Vehicle Route in the week**	**Cap**
A_1	C → 15 → D → C	3	C → 15 → D → C	3
A_2	C→ 1 → 9 → 3 → 7 → 8→ D → C	2.982	C→ 1 → 9 → 7 → 8→ D → C	2.241
A_3	C → 4 → 5 → 10 → 11 → 12 → D → C	2.948	C → 4 → 5 → 10 → 11 → D → C	2.809
A_4	C→ 2 → 6 → 14 → 13 → D → C	2.762	C→ 2 → 6 → 14 → 13 → D → C	2.558
A_5	------------------	0	C → 12 → 8 → D → C	1.252

TABLE 5 Shortest route for each vehicle using CDQN

No.	Vehicle Route in the week	Cap	Vehicle Route in the week	Cap
A_1	C → 15 → D → C	3	C → 15 → D → C	3
A_2	C→ 2 → 5 → 11 → 12 → D → C	2.922	C→ 2 → 5 → 11 → D → C	2.641
A_3	C → 10 → 3 → 6 → 7 → 8 → 13 → D → C	2.874	C → 10 → 3 → 6 → 7 → 8 → 13 → D → C	2.68
A_4	C → 4 → 1 → 9 → 14 → D → C	2.896	C → 4 → 1 → 9 → D → C	2.409
A_5	------------------	0	C → 12 → 14→ D → C	1.13

TABLE 6 Shortest route for each vehicle using FKPDQN

No.	Vehicle Route in the week	Cap	Vehicle Route in the week	Cap
A_1	C → 15 → D → C	3	C → 15 → D → C	3
A_2	C→ 10 → 7 → 6 → 3 →13 → 11→ 9 → 4 → D → C	3	C→ 10 → 7 → 6 → 3 →13 → 11→ 9 → 4 → D → C	3
A_3	C → 4 → 5 → 8 → 2 → D → C	3	C → 4 → 5 → 8 → 2 → D → C	3
A_4	C → 2 → 12 → 1 → 14 → D → C	2.692	C → 2 → 12 → 1 → 14 → D → C	2.860

TABLE 7 Percentage Comparison Based on Reference (Optimal) Values

Metric	CQL	A*	DQN	A* with DQN	CDQN	Fractional Knapsack with DQN (FKPDQN)
**Number of Vehicles**	7 (42.86% increase)	5 (20% increase)	5 (20% increase)	5 (20% increase)	5 (20% increase)	4 (0% loss)
**Vehicle Capacity Utilization**	33% (-67%)	66% (-34%)	66% (-34%)	50% (-50%)	60% (-40%)	100% (optimal)
**Distance**	622.43 km (+17.5%)	529.6 km (reference)	609.2 km (+15%)	588.3 km (+11%)	599.43 km (+13%)	580.2 km (+9%)
**Time**	1006 min (+19%)	841 min (reference)	912 min (+8.5%)	949 min (+12.8%)	940 min (+11.8%)	913 min (+8.5%)
**Fuel Cost**	$20.39 per liter (+17%)	$17.35 per liter (reference)	$19.96 per liter (+15%)	$19.27 per liter (+11%)	$19.64 per liter (+13%)	$19.01 per liter (+9%)
**Battery Cost**	$2.61 per unit (+15%)	$2.22 per unit (reference)	$2.55 per unit (+15%)	$2.47 per unit (+11%)	$2.51 per unit (+13%)	$2.43 per unit (+9%)

2 Supplementary Figures

The figures related to the issue were collected separately and are used to support and analyze the data presented in the study.

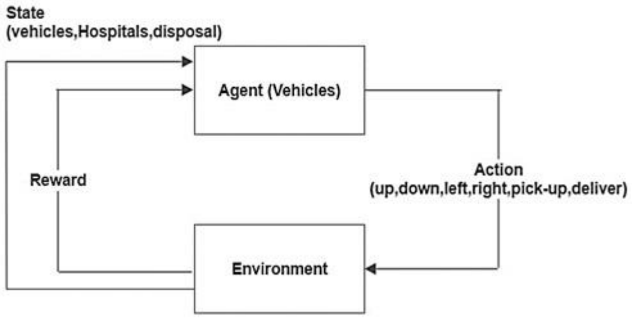

FIGURE 1

RL framework for heterogeneous CVRP

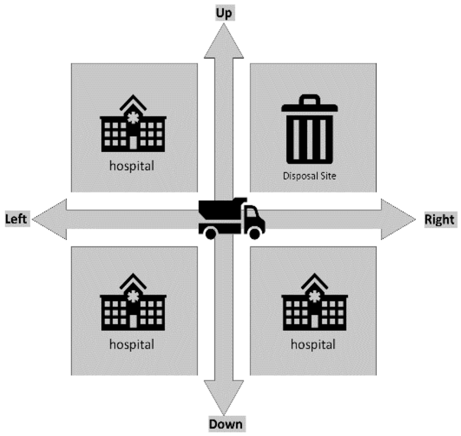

FIGURE 2

Flowchart of State inside matrix

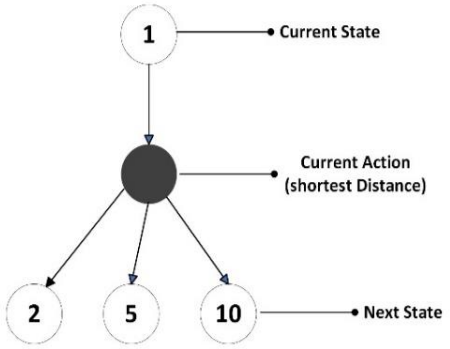

FIGURE 3

Relationship State and Actions

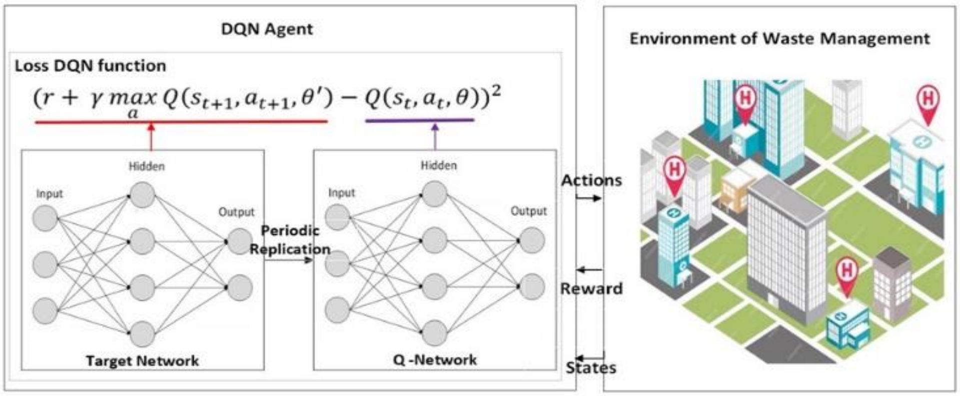

FIGURE 4

A diagram of deep Q network algorithm

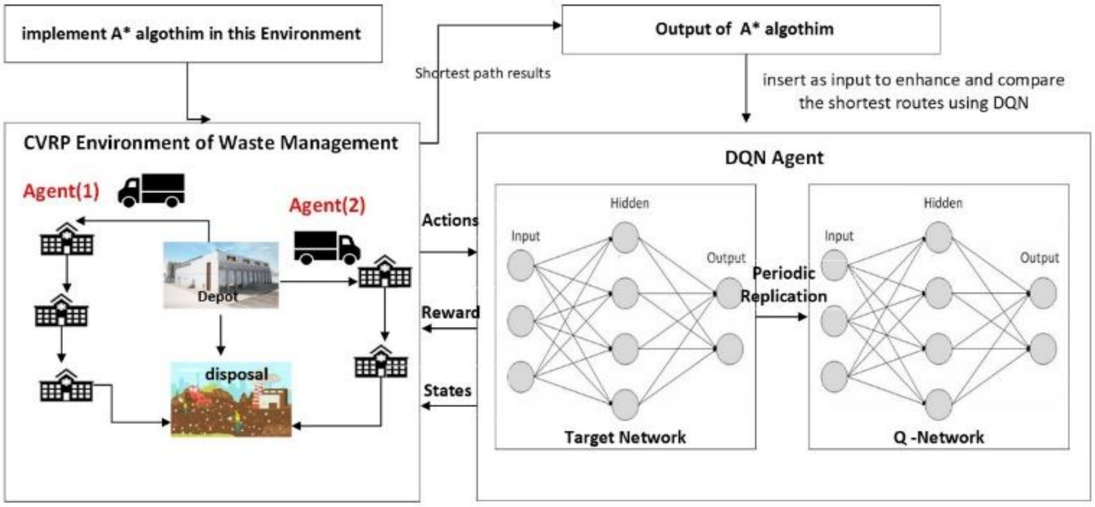

FIGURE 5

A Diagram of hybridization between A* and DQN

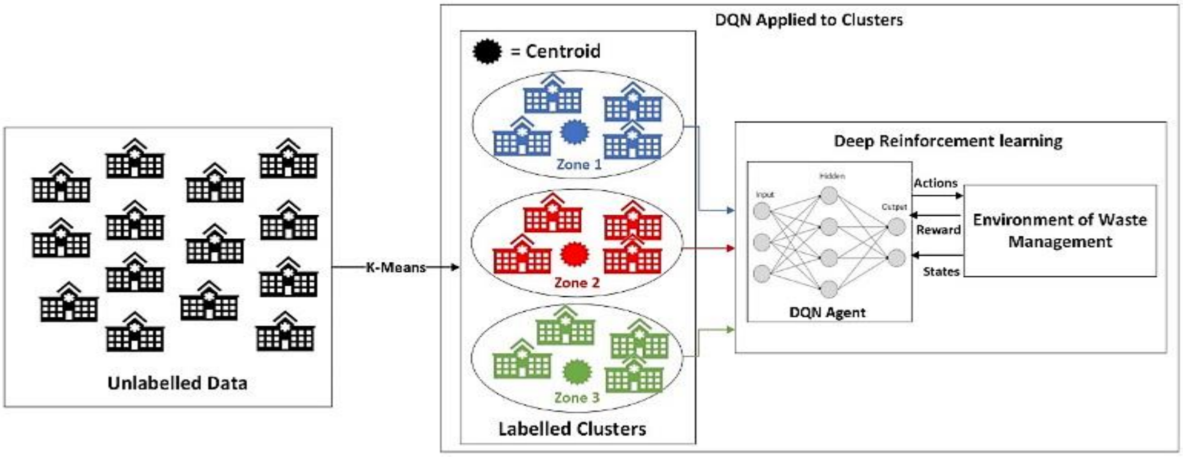

FIGURE 6

Diagram of K-means and DQN Hybridization

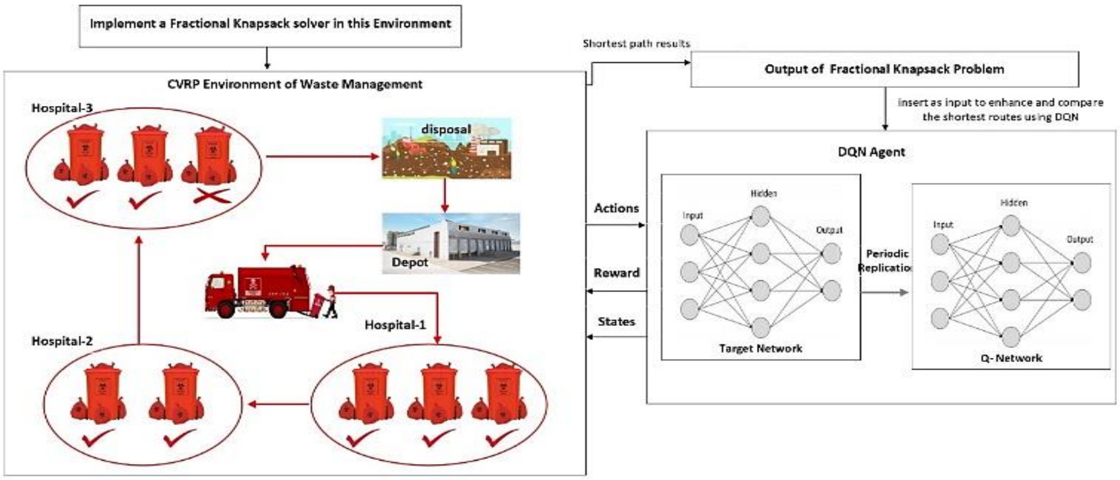

FIGURE 7

Diagram of FKP and DQN Hybridization

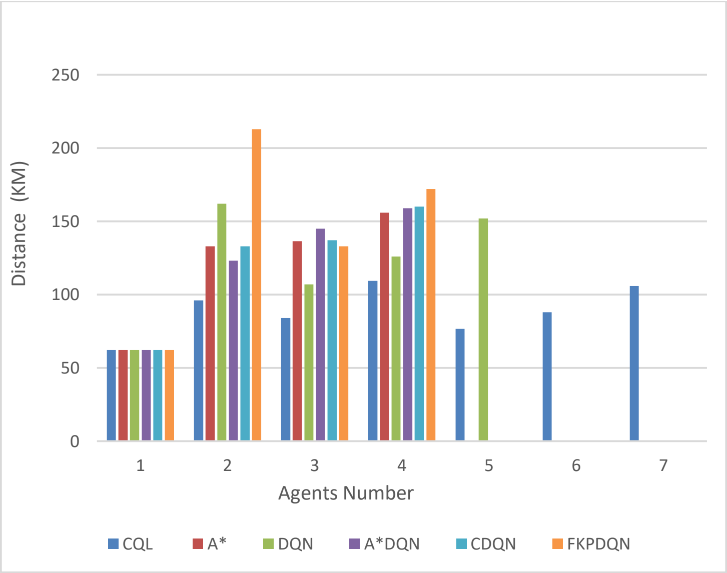

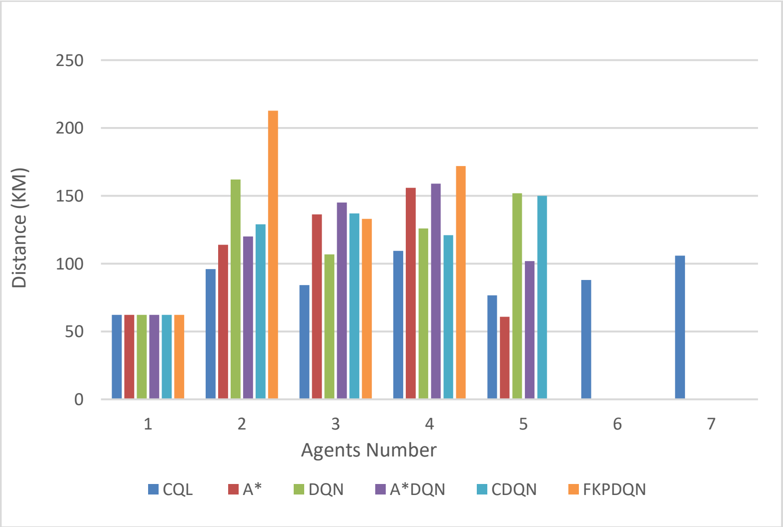

FIGURE 9

Estimated distance per agent during 2nd visit

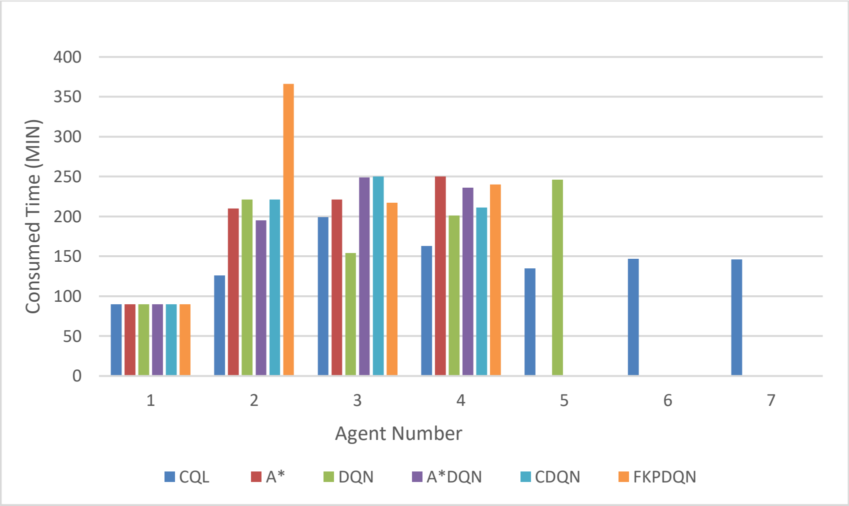

FIGURE 10

Time spent per agent during the 1st visit

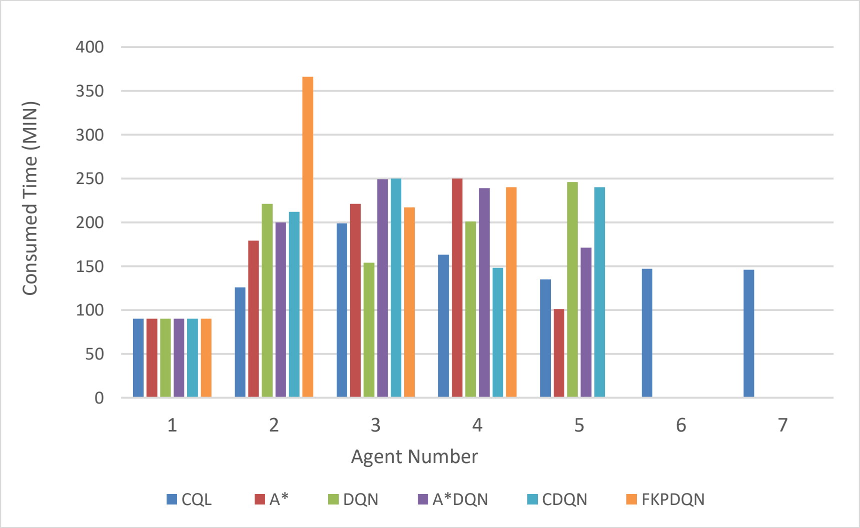

FIGURE 11

Time spent per agent during the 2nd visit

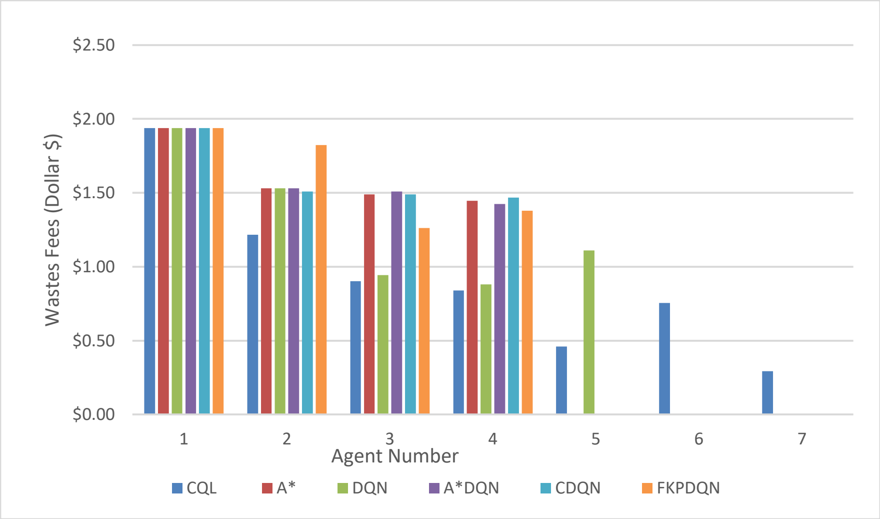

FIGURE 12

Fees of hospitals wastes during $\mathbf{1}^{st}$ visit

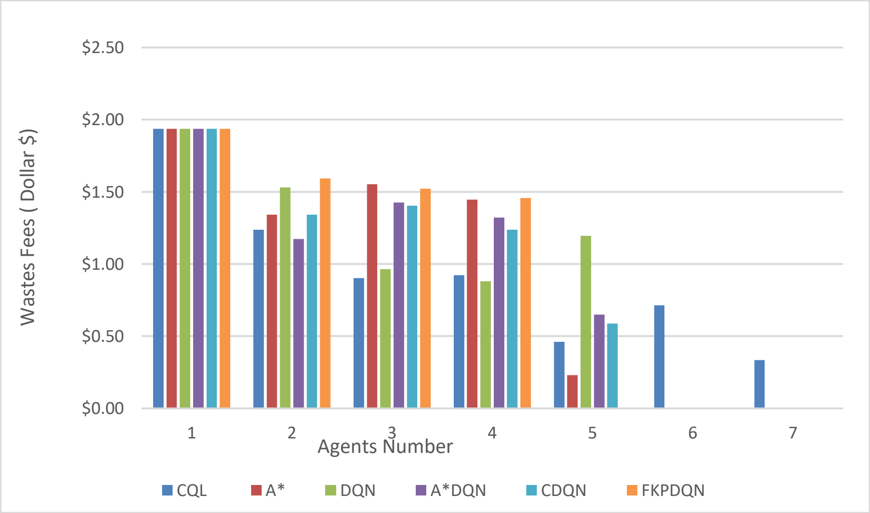

FIGURE 13

Fees of hospitals wastes during $\mathbf{2}^{nd}$ visit

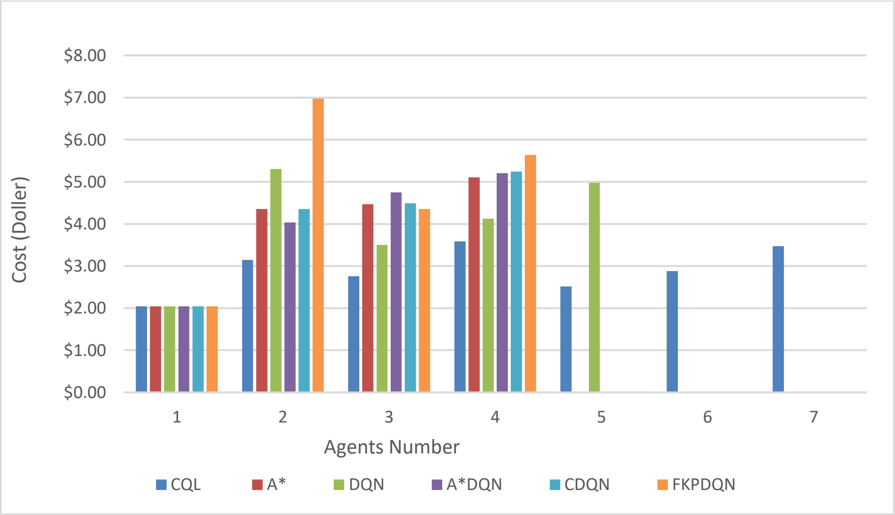

FIGURE 14

Transportation cost by using fuel during 1st visit

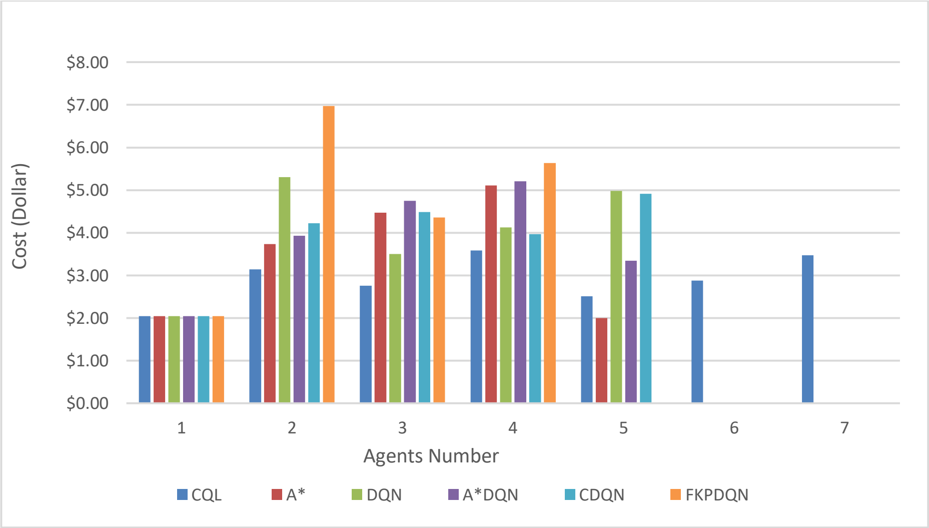

FIGURE 15

Transportation cost by using fuel during 2nd visit

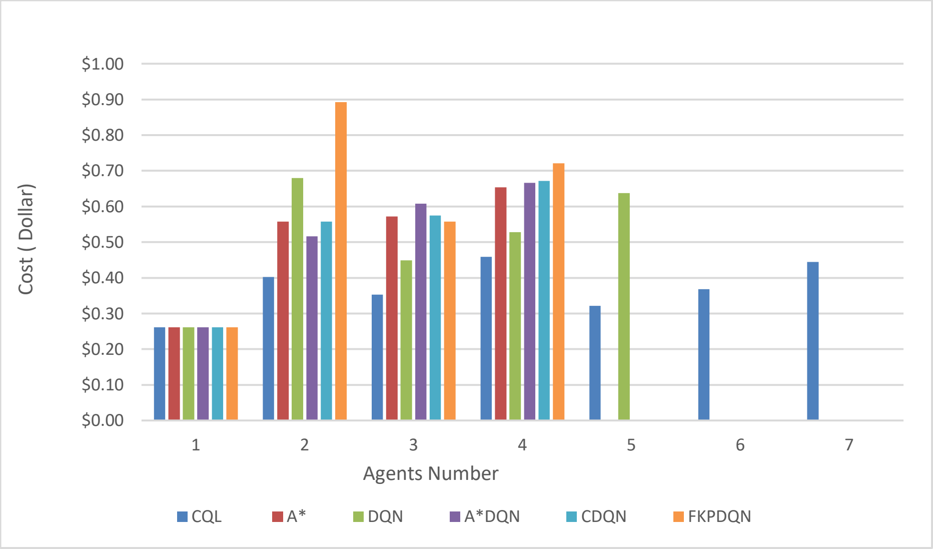

FIGURE 16

Transportation cost by Electric agents during 1st visit

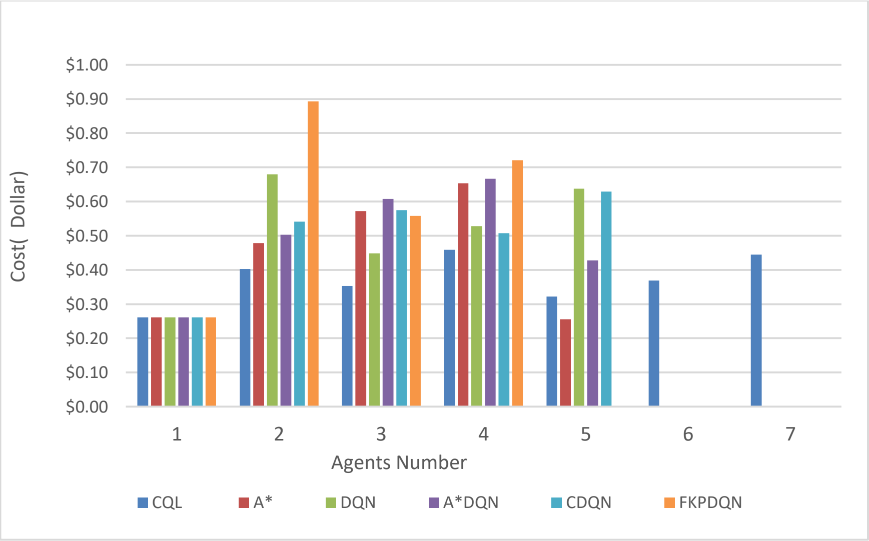

FIGURE 17

Transportation cost by Electric agents during 2nd visit
